# Crystal structures of (*E*)-3-(furan-2-yl)-2-phenyl-*N*-tosyl­acryl­amide and (*E*)-3-phenyl-2-(*m*-tol­yl)-*N*-tosyl­acryl­amide

**DOI:** 10.1107/S2056989016007611

**Published:** 2016-05-10

**Authors:** Dong Cheng, Xiangzhen Meng, Zeyuan Sheng, Shuangming Wang, Yuanyuan Duan, Ziqian Li

**Affiliations:** a School of Chemistry and Materials Engineering, Chaohu College, Chaohu Anhui, People’s Republic of China

**Keywords:** crystal structure, Cu-catalysed azide-alkyne cyclo­addition reaction, CuAAC, N—H⋯O hydrogen bonding, inversion dimers, C—H⋯π inter­actions

## Abstract

In the title *N*-tosyl­acryl­amide compounds, C_20_H_17_NO_4_S, (I), and C_23_H_21_NO_3_S, (II), the conformation about the C=C bond is *E*. The acryl­amide groups, [–NH—C(=O)—C=C–], are almost planar, with the N—C—C=C torsion angle being −170.18 (14)° in (I) and −168.01 (17)° in (II). In (I), the furan, phenyl and 4-methyl­benzene rings are inclined to the acryl­amide mean plane by 26.47 (11), 69.01 (8) and 82.49 (9)°, respectively. In (II), the phenyl, 3-methyl­benzene and 4-methyl­benzene rings are inclined to the acryl­amide mean plane by 11.61 (10), 78.44 (10) and 78.24 (10)°, respectively. There is an intra­molecular C—H⋯π inter­action present in compound (II). In the crystals of both compounds, mol­ecules are linked by pairs of N—H⋯O hydrogen bonds, forming inversion dimers with an *R*_2_^2^(8) ring motif. In (I), the dimers are reinforced by C—H⋯O hydrogen bonds and linked by C—H⋯π inter­actions, forming chains along [011]. In the crystal of (II), the dimers are linked *via* C—H⋯O hydrogen bonds, forming chains along [100]. The chains are further linked by C—H⋯π inter­actions, forming layers parallel to (010).

## Abstract 2

In the title *N*-tosyl­acryl­amide compounds, (I) and (II), the conformation about the C=C bond is *E*. In (I), the furan, phenyl and 4-methyl­benzene rings are inclined to the acryl­amide mean plane [–NH—C(= O)—C=C–] by 26.47 (11), 69.01 (8) and 82.49 (9)°, respectively. In (II), the phenyl, and 3-methyl and 4-methyl­benzene rings are inclined to the acryl­amide mean plane by 11.61 (10), 78.44 (10) and 78.24 (10)°, respectively. In the crystals of both compounds, mol­ecules are linked by pairs of N—H⋯O hydrogen bonds, forming inversion dimers with 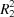
(8) ring motifs.

## Chemical context   

The Cu-catalysed azide-alkyne cyclo­addition (CuAAC) reaction constitutes one of the most inter­esting examples of the click reaction (Bae *et al.*, 2005 ▸; Cheng *et al.*, 2012 ▸; Mondal & Pan, 2015 ▸). Trisubstituted alkenes are commonly found in the mol­ecular skeleton of natural products and bioactive substances, and they are important building blocks in organic chemistry (Zhu *et al.*, 2012 ▸; Manikandan & Jeganmohan, 2015 ▸). Therefore, it is highly desirable to develop new efficient and general methods for the stereoselective synthesis of tris­ubstituted alkenes (Ram & Tittal, 2014 ▸; Bae *et al.*, 2005 ▸). As part of our work on the application of the CuAAC reaction (Cheng *et al.*, 2012 ▸), we report herein on the synthesis and crystal structures of the title compounds, (I) and (II).

## Structural commentary   

**Figure 1:**
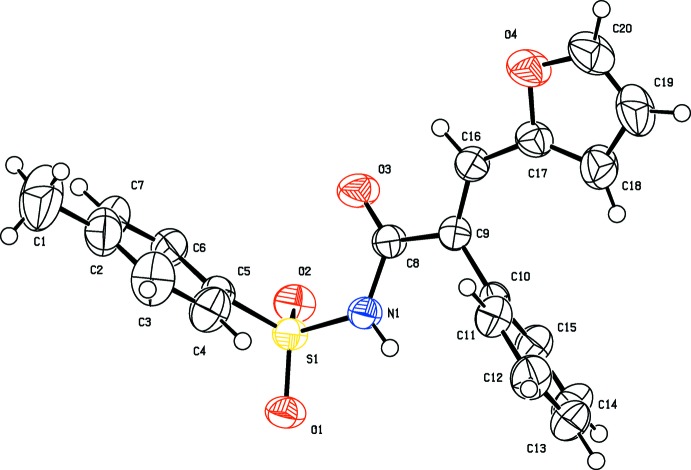
The mol­ecular structure of compound (I), showing the atom labelling and displacement ellipsoids drawn at the 50% probability level.

The mol­ecular structures of the title compounds, (I) and (II), are illustrated in Figs. 1 ▸ and 2 ▸, respectively. Both mol­ecules adopt an *E* conformation about the C=C bonds; C9=C16 in (I) and C9=C10 in (II). The acryl­amide groups, [–NH—C(=O)—C=C–], are almost planar with the N1—C8—C9=C16 torsion angle being −170.18 (14) ° in (I), and the N1—C8—C9=C10 torsion angle being −168.01 (17)° in (II). The mol­ecular conformation of the two mol­ecules differ somewhat, as shown by the structure overlap illustrated in Fig. 3 ▸.
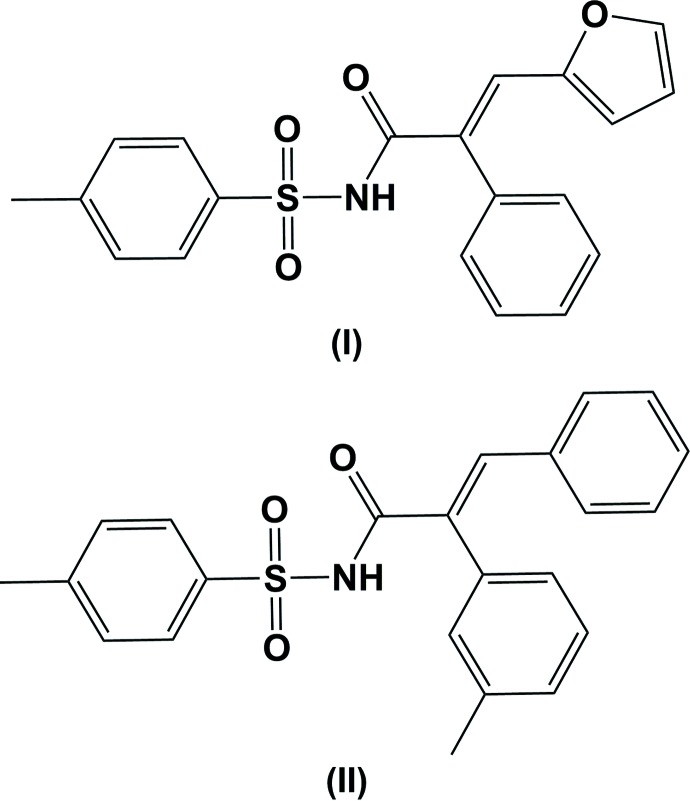


**Figure 2:**
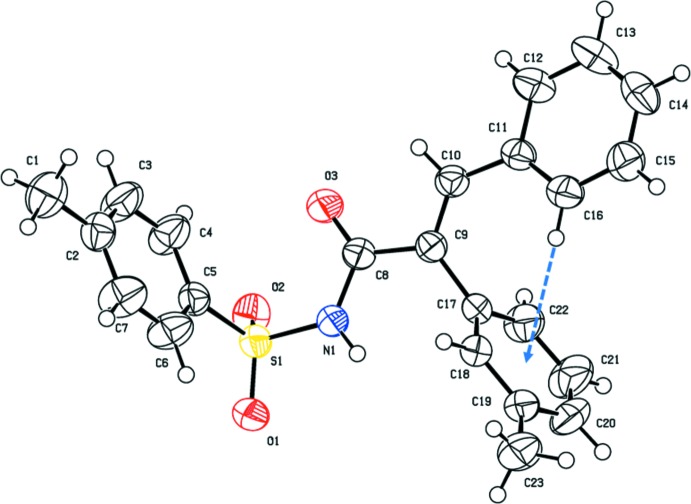
The mol­ecular structure of compound (II), showing the atom labelling and displacement ellipsoids drawn at the 50% probability level. The intra­molecular C—H⋯π inter­action is shown by the blue dashed arrow (see Table 2 ▸).

**Figure 3:**
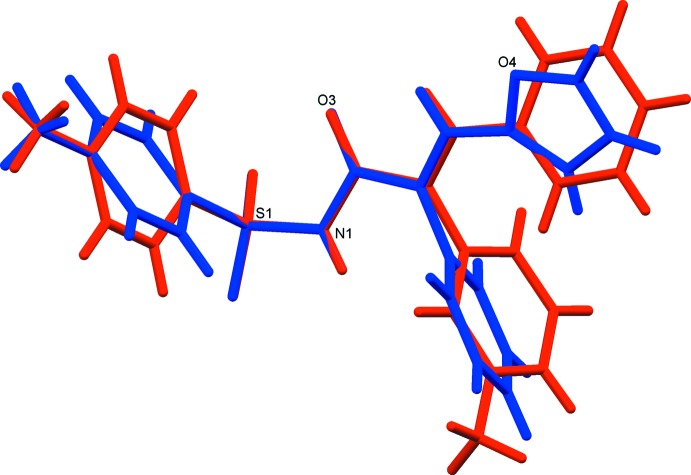
A view of the overlap of mol­ecules (I) (blue) and (II) (red).

 In (I) the furan, phenyl and 4-methyl­benzene rings are inclined to the acryl­amide mean plane [N1/O3/C8/C9/C16; maximum deviation of 0.0779 (15) Å for atom C9] by 26.47 (11), 69.01 (8) and 82.49 (9)°, respectively. The 4-methyl­benzene ring is inclined to the furan and phenyl rings by 72.25 (11) and 19.00 (9)°, respectively, the latter two rings being inclined to one another by 66.28 (11)°. In (II), the phenyl, 3-methyl­benzene and 4-methyl­benzene rings are inclined to the acryl­amide mean plane [N1/O3/C8/C9/C10; maximum deviation of 0.0998 (18) Å for atom C9] by 11.61 (10), 78.44 (10) and 78.24 (10)°, respectively. The 4-methyl­benzene ring is inclined to the phenyl and 3-methyl­benzene rings by dihedral angles of 78.33 (11) and 13.10 (11)°, respectively, the latter two rings being inclined to one another by 75.86 (11)°. There is an intra­molecular C—H⋯π inter­action present in compound (II) involving the adjacent phenyl and 3-methyl­benzene rings (Table 2 and Fig. 2 ▸).

## Supra­molecular features   

**Figure 4:**
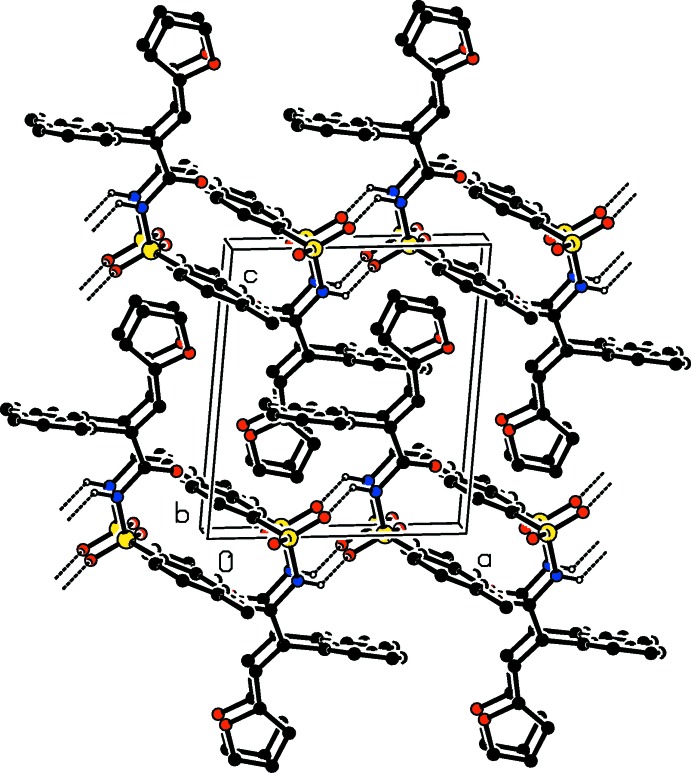
The crystal packing of compound (I), viewed along the *b*-axis direction. The hydrogen bonds are shown as dashed lines (see Table 1 ▸), and for clarity only the H atoms involved in the various inter­actions have been included.

**Figure 5:**
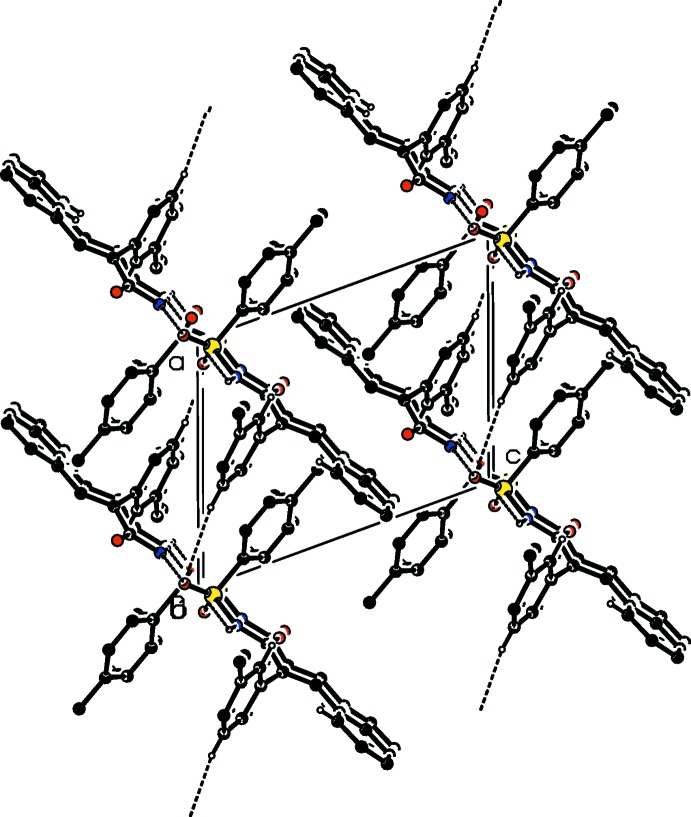
The crystal packing of compound (II), viewed along the *b*-axis direction. The hydrogen bonds are shown as dashed lines (see Table 2 ▸), and for clarity only the H atoms involved in the various inter­actions have been included.

In the crystal of both compounds, mol­ecules are linked by pairs of N—H⋯O hydrogen bonds (Tables 1 ▸ and 2 ▸), forming inversion dimers with 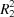
(8) ring motifs, as shown in Fig. 4 ▸ for (I) and Fig. 5 ▸ for (II). In (I), the dimers are reinforced by C—H⋯O hydrogen bonds and linked by C—H⋯π inter­actions (Table 1 ▸), forming chains propagating along [011]. In the crystal of (II), the dimers are linked *via* C—H⋯O hydrogen bonds, forming chains propagating along [100]. There is also a C—H⋯π inter­action present, linking the chains to form layers lying parallel to (010).

## Database survey   

A search of the Cambridge Structural Database (Version 5.37, update February 2016; Groom *et al.*, 2016 ▸) for the substructure *N*-(phenyl­sulfon­yl)acryl­amide yielded five hits. Four of these compounds involve the 4-methyl­benzene­sulfonyl group and one compound involves a phenyl­sulfonyl group. This later compound, 2-(4-chloro­phen­yl)-3-(2-fur­yl)-*N*-(phenyl­sulfon­yl)acryl­amide (BIZGOI; Yu & Cao, 2014 ▸), is very similar to compound (I). The principal difference in the conformation of this mol­ecule with respect to that of compound (I) is the dihedral angle involving the pyran ring and the adjacent aromatic ring, a phenyl ring in (I) and a chloro­benzene ring in BIZGOI; this angle is 66.18 (11)° in (I) but 88.84 (13)° in BIZGOI. In the crystal of BIZGOI, mol­ecules are linked by pairs of N—H⋯O hydrogen bonds, forming inversion dimers with an 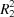
(8) ring motif, similar to the arrangement in the crystals of compounds (I) and (II).

## Synthesis and crystallization   

**Compound (I)**: 4-methyl­benzene­sulfonyl azide (4.5 mmol), CuI (5.7 mg, 0.03 mmol), Et_4_NI (7.7 mg, 0.03 mmol), ethynyl­benzene (4.5 mmol), and furan-2-carbaldehyde (3 mmol) were suspended in CH_2_Cl_2_ (5 ml) in a 10 mL Schlenk tube under nitro­gen at rt. LiOH (8.64 mg, 3.6mmol) was then added, and the resulting solution was stirred at this temperature. Upon full consumption of furan-2-carbaldehyde, the reaction was quenched by saturated aqueous NH_4_Cl (5 ml) and extracted with CH_2_Cl_2_ (10 ml × 3). The combined organic layers were dried over anhydrous Na_2_SO_4_ and concentrated in vacuo. The crude residue was purified by column chroma­tography on silica gel (*n*-hexa­ne/EtOAc 5:1 *v*/*v*) to afford compound (I) as a white solid (yield: 0.79 g, 72%). Part of the purified product was redissolved in *n*-hexa­ne/EtOAc and after slow evaporation over several days, colourless crystals suitable for analysis by X-ray diffraction were formed.

**Compound (II)**: 4-methyl­benzene­sulfonyl azide (4.5 mmol), CuI (5.7 mg, 0.03 mmol), Et_4_NI (7.7 mg, 0.03 mmol), 1-eth­yn­yl-3-methyl­benzene (4.5 mmol), and benzaldehyde (3 mmol) were suspended in CH_2_Cl_2_ (5 ml) in a 10 mL Schlenk tube under nitro­gen at rt. LiOH (8.64 mg, 3.6mmol) was then added, and the resulting solution was stirred at this temperature. Upon full consumption of benzaldehyde, the reaction was quenched by saturated aqueous NH_4_Cl (5 ml) and extracted with CH_2_Cl_2_ (3 × 10 ml). The combined organic layers were dried over anhydrous Na_2_SO_4_ and concentrated *in vacuo*. The crude residue was purified by column chroma­tography on silica gel (*n*-hexa­ne/EtOAc 5:1 *v*/*v*) to afford compound (II) as a white solid (0.82, 70%). Part of the purified product was redissolved in *n*-hexa­ne/EtOAc and after slow evaporation over several days, colourless block-like crystals were obtained.

## Refinement   

Crystal data, data collection and structure refinement details are summarized in Table 3 ▸. H atoms were placed in geom­etrically idealized positions and constrained to ride on their parent atoms: C—H = 0.93–0.96 Å and N—H = 0.86 Å, with *U*_iso_(H) = 1.5*U*_eq_(C-meth­yl) and 1.2*U*_eq_(C,N) for other H atoms.

## Supplementary Material

Crystal structure: contains datablock(s) global, I, II. DOI: 10.1107/S2056989016007611/su5296sup1.cif

Supporting information file. DOI: 10.1107/S2056989016007611/su5296Isup2.cml

Supporting information file. DOI: 10.1107/S2056989016007611/su5296IIsup3.cml

CCDC references: 1478730, 1478729

Additional supporting information:  crystallographic information; 3D view; checkCIF report

## Figures and Tables

**Table 1:** Hydrogen-bond geometry (Å, °) for (I) *Cg*1 is the centroid of the furan ring, O4/C17–C20

*D*—H⋯*A*	*D*—H	H⋯*A*	*D*⋯*A*	*D*—H⋯*A*
N1—H1⋯O1^i^	0.86	2.30	2.904 (2)	127
C4—H4⋯O1^i^	0.93	2.55	3.427 (3)	158
C12—H12⋯*Cg*1^ii^	0.93	2.81	3.664 (2)	158

Symmetry codes: (i) 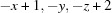; (ii) 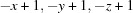.

**Table 2:** Hydrogen-bond geometry (Å, °) for (II) *Cg*2 and *Cg*3 are the centroids of rings C11–C16 and C17–C22, respectively.

*D*—H⋯*A*	*D*—H	H⋯*A*	*D*⋯*A*	*D*—H⋯*A*
N1—H1⋯O1^i^	0.86	2.31	3.038 (2)	143
C21—H21⋯O2^ii^	0.93	2.57	3.468 (4)	163
C16—H16⋯*Cg*3	0.93	2.88	3.617 (2)	137
C18—H18⋯*Cg*2^iii^	0.93	2.83	3.646 (2)	168

Symmetry codes: (i) 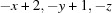; (ii) 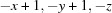; (iii) 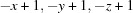.

**Table 3:** Experimental details

	(I)	(II)
Crystal data		
Chemical formula	C_20_H_17_NO_4_S	C_23_H_21_NO_3_S
*M*_r_	367.41	391.47
Crystal system, space group	Triclinic, *P* 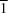	Triclinic, *P* 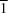
Temperature (K)	293	293
*a*, *b*, *c* (Å)	10.309 (2), 10.391 (2), 10.566 (2)	9.2595 (10), 10.1158 (11), 11.9271 (12)
α, β, γ (°)	69.598 (2), 75.790 (2), 61.445 (2)	72.396 (1), 67.518 (1), 79.346 (1)
*V* (Å^3^)	927.5 (3)	980.89 (18)
*Z*	2	2
Radiation type	Mo *K*α	Mo *K*α
μ (mm^−1^)	0.20	0.19
Crystal size (mm)	0.21 × 0.20 × 0.19	0.23 × 0.22 × 0.19

Data collection		
Diffractometer	Bruker APEXII CCD area-detector	Bruker *SMART* CCD area-detector
Absorption correction	Multi-scan (*SADABS*; Bruker, 2008 ▸)	Multi-scan (*SADABS*; Bruker, 2008 ▸)
*T*_min_, *T*_max_	0.959, 0.963	0.958, 0.965
No. of measured, independent and observed [*I* > 2σ(*I*)] reflections	8964, 3258, 3012	7136, 3422, 3082
*R*_int_	0.024	0.020
(sin θ/λ)_max_ (Å^−1^)	0.595	0.595

Refinement		
*R*[*F* ^2^ > 2σ(*F* ^2^)], *wR*(*F* ^2^), *S*	0.035, 0.102, 1.04	0.040, 0.103, 1.00
No. of reflections	3258	3422
No. of parameters	237	255
H-atom treatment	H-atom parameters constrained	H-atom parameters constrained
Δρ_max_, Δρ_min_ (e Å^−3^)	0.30, −0.31	0.24, −0.35

Computer programs: *APEX2* and *SAINT* (Bruker, 2008 ▸), *SHELXS97*, *SHELXL97* and *SHELXTL* (Sheldrick, 2008 ▸), *PLATON* (Spek, 2009 ▸) and *Mercury* (Macrae *et al.*, 2008 ▸).

## supplementary crystallographic information

### Crystal data

**Table:**

C_23_H_21_NO_3_S	*Z* = 2
*M**_r_* = 391.47	*F*(000) = 412
Triclinic, *P*1	*D*_x_ = 1.325 Mg m^−^^3^
Hall symbol: -P 1	Mo *K*α radiation, λ = 0.71073 Å
*a* = 9.2595 (10) Å	Cell parameters from 5782 reflections
*b* = 10.1158 (11) Å	θ = 2.4–27.5°
*c* = 11.9271 (12) Å	µ = 0.19 mm^−^^1^
α = 72.396 (1)°	*T* = 293 K
β = 67.518 (1)°	Block, colorless
γ = 79.346 (1)°	0.23 × 0.22 × 0.19 mm
*V* = 980.89 (18) Å^3^	

### Data collection

**Table:**

Bruker SMART CCD area-detector diffractometer	3422 independent reflections
Radiation source: fine-focus sealed tube	3082 reflections with *I* > 2σ(*I*)
Graphite monochromator	*R*_int_ = 0.020
Detector resolution: 18.4 pixels mm^-1^	θ_max_ = 25.0°, θ_min_ = 1.9°
phi and ω scans	*h* = −11→11
Absorption correction: multi-scan (*SADABS*; Bruker, 2008)	*k* = −11→12
*T*_min_ = 0.958, *T*_max_ = 0.965	*l* = −13→14
7136 measured reflections	

### Refinement

**Table:**

Refinement on *F*^2^	Primary atom site location: structure-invariant direct methods
Least-squares matrix: full	Secondary atom site location: difference Fourier map
*R*[*F*^2^ > 2σ(*F*^2^)] = 0.040	Hydrogen site location: inferred from neighbouring sites
*wR*(*F*^2^) = 0.103	H-atom parameters constrained
*S* = 1.00	*w* = 1/[σ^2^(*F*_o_^2^) + (0.0455*P*)^2^ + 0.4517*P*] where *P* = (*F*_o_^2^ + 2*F*_c_^2^)/3
3422 reflections	(Δ/σ)_max_ = 0.020
255 parameters	Δρ_max_ = 0.24 e Å^−^^3^
0 restraints	Δρ_min_ = −0.35 e Å^−^^3^

### Special details

**Table:**

Geometry. Bond distances, angles etc. have been calculated using the rounded fractional coordinates. All su's are estimated from the variances of the (full) variance-covariance matrix. The cell esds are taken into account in the estimation of distances, angles and torsion angles
Refinement. Refinement on F^2^ for ALL reflections except those flagged by the user for potential systematic errors. Weighted R-factors wR and all goodnesses of fit S are based on F^2^, conventional R-factors R are based on F, with F set to zero for negative F^2^. The observed criterion of F^2^ > 2sigma(F^2^) is used only for calculating -R-factor-obs etc. and is not relevant to the choice of reflections for refinement. R-factors based on F^2^ are statistically about twice as large as those based on F, and R-factors based on ALL data will be even larger.

### Fractional atomic coordinates and isotropic or equivalent isotropic displacement parameters (Å^2^)

**Table:**

	*x*	*y*	*z*	*U*_iso_*/*U*_eq_	
S1	0.95790 (5)	0.26449 (4)	0.05551 (4)	0.0436 (1)	
O1	1.04900 (15)	0.37065 (13)	−0.04088 (12)	0.0547 (4)	
O2	0.90276 (16)	0.16492 (14)	0.02290 (13)	0.0569 (5)	
O3	0.68817 (16)	0.16464 (13)	0.28061 (14)	0.0597 (5)	
N1	0.80466 (16)	0.35319 (15)	0.13653 (14)	0.0451 (5)	
C1	1.3053 (3)	−0.0307 (3)	0.4111 (2)	0.0727 (9)	
C2	1.2199 (2)	0.0429 (2)	0.32171 (19)	0.0528 (6)	
C3	1.1116 (3)	−0.0221 (2)	0.3086 (3)	0.0722 (8)	
C4	1.0318 (3)	0.0434 (2)	0.2278 (2)	0.0675 (8)	
C5	1.05970 (19)	0.17789 (17)	0.15828 (17)	0.0428 (5)	
C6	1.1700 (3)	0.2442 (2)	0.1675 (2)	0.0633 (8)	
C7	1.2484 (3)	0.1761 (2)	0.2490 (2)	0.0693 (8)	
C8	0.68331 (19)	0.28973 (18)	0.24058 (16)	0.0428 (5)	
C9	0.55006 (18)	0.38643 (17)	0.29569 (16)	0.0386 (5)	
C10	0.44805 (19)	0.32908 (18)	0.40848 (16)	0.0426 (5)	
C11	0.3048 (2)	0.39036 (19)	0.48921 (16)	0.0429 (5)	
C12	0.1949 (2)	0.2998 (2)	0.57903 (18)	0.0562 (7)	
C13	0.0586 (3)	0.3480 (3)	0.6607 (2)	0.0681 (8)	
C14	0.0297 (2)	0.4866 (3)	0.6551 (2)	0.0658 (8)	
C15	0.1372 (3)	0.5781 (2)	0.5689 (2)	0.0640 (7)	
C16	0.2740 (2)	0.5309 (2)	0.48597 (18)	0.0543 (6)	
C17	0.52982 (18)	0.53233 (17)	0.22211 (15)	0.0394 (5)	
C18	0.60582 (19)	0.63895 (17)	0.22063 (16)	0.0419 (5)	
C19	0.5787 (2)	0.77626 (19)	0.15814 (18)	0.0526 (6)	
C20	0.4696 (3)	0.8034 (2)	0.0996 (2)	0.0756 (8)	
C21	0.3957 (3)	0.6992 (3)	0.0975 (3)	0.0837 (10)	
C22	0.4264 (2)	0.5634 (2)	0.1573 (2)	0.0606 (7)	
C23	0.6632 (3)	0.8906 (2)	0.1559 (2)	0.0737 (8)	
H1	0.80020	0.44260	0.11300	0.0540*	
H1A	1.23480	−0.03780	0.49580	0.1090*	
H1B	1.39150	0.02090	0.39490	0.1090*	
H1C	1.34430	−0.12220	0.39960	0.1090*	
H3	1.09170	−0.11290	0.35580	0.0870*	
H4	0.95940	−0.00300	0.22020	0.0810*	
H6	1.19150	0.33430	0.11900	0.0760*	
H7	1.32280	0.22150	0.25500	0.0830*	
H10	0.47200	0.23520	0.44070	0.0510*	
H12	0.21380	0.20500	0.58400	0.0670*	
H13	−0.01370	0.28600	0.71960	0.0820*	
H14	−0.06280	0.51920	0.70970	0.0790*	
H15	0.11800	0.67230	0.56630	0.0770*	
H16	0.34570	0.59370	0.42760	0.0650*	
H18	0.67690	0.61820	0.26250	0.0500*	
H20	0.44570	0.89480	0.06040	0.0910*	
H21	0.32450	0.72020	0.05570	0.1000*	
H22	0.37780	0.49260	0.15420	0.0730*	
H23A	0.74480	0.91580	0.07570	0.1100*	
H23B	0.70830	0.85900	0.22060	0.1100*	
H23C	0.59060	0.97000	0.16980	0.1100*	

### Atomic displacement parameters (Å^2^)

**Table:**

	*U*^11^	*U*^22^	*U*^33^	*U*^12^	*U*^13^	*U*^23^
S1	0.0405 (2)	0.0405 (2)	0.0452 (3)	−0.0018 (2)	−0.0106 (2)	−0.0112 (2)
O1	0.0506 (7)	0.0506 (7)	0.0466 (7)	−0.0047 (6)	−0.0035 (6)	−0.0068 (6)
O2	0.0592 (8)	0.0539 (8)	0.0666 (9)	−0.0005 (6)	−0.0276 (7)	−0.0231 (7)
O3	0.0540 (8)	0.0372 (7)	0.0687 (9)	−0.0055 (6)	−0.0057 (7)	−0.0057 (6)
N1	0.0395 (8)	0.0350 (7)	0.0501 (9)	−0.0023 (6)	−0.0074 (6)	−0.0071 (6)
C1	0.0784 (15)	0.0720 (15)	0.0750 (15)	0.0034 (12)	−0.0401 (13)	−0.0171 (12)
C2	0.0521 (11)	0.0516 (11)	0.0550 (11)	0.0029 (8)	−0.0188 (9)	−0.0178 (9)
C3	0.0748 (14)	0.0441 (11)	0.0995 (18)	−0.0114 (10)	−0.0474 (14)	0.0052 (11)
C4	0.0659 (13)	0.0444 (11)	0.0996 (17)	−0.0145 (9)	−0.0470 (13)	0.0009 (11)
C5	0.0381 (9)	0.0391 (9)	0.0479 (10)	−0.0021 (7)	−0.0104 (7)	−0.0132 (7)
C6	0.0806 (15)	0.0465 (11)	0.0705 (14)	−0.0199 (10)	−0.0357 (12)	−0.0049 (10)
C7	0.0858 (16)	0.0602 (13)	0.0792 (15)	−0.0231 (11)	−0.0462 (13)	−0.0091 (11)
C8	0.0392 (9)	0.0405 (9)	0.0462 (10)	−0.0066 (7)	−0.0135 (7)	−0.0072 (8)
C9	0.0353 (8)	0.0389 (9)	0.0424 (9)	−0.0058 (7)	−0.0150 (7)	−0.0080 (7)
C10	0.0421 (9)	0.0403 (9)	0.0451 (10)	−0.0073 (7)	−0.0159 (8)	−0.0072 (7)
C11	0.0410 (9)	0.0501 (10)	0.0359 (9)	−0.0088 (7)	−0.0120 (7)	−0.0075 (7)
C12	0.0557 (11)	0.0594 (12)	0.0504 (11)	−0.0194 (9)	−0.0073 (9)	−0.0154 (9)
C13	0.0538 (12)	0.0868 (16)	0.0556 (12)	−0.0284 (11)	0.0036 (10)	−0.0227 (11)
C14	0.0470 (11)	0.0891 (17)	0.0547 (12)	−0.0032 (11)	−0.0042 (9)	−0.0280 (12)
C15	0.0663 (13)	0.0598 (12)	0.0554 (12)	0.0057 (10)	−0.0132 (10)	−0.0167 (10)
C16	0.0537 (11)	0.0515 (11)	0.0453 (10)	−0.0060 (9)	−0.0068 (9)	−0.0074 (8)
C17	0.0324 (8)	0.0425 (9)	0.0369 (8)	−0.0024 (7)	−0.0082 (7)	−0.0067 (7)
C18	0.0389 (9)	0.0418 (9)	0.0403 (9)	−0.0023 (7)	−0.0119 (7)	−0.0067 (7)
C19	0.0506 (10)	0.0417 (10)	0.0511 (11)	−0.0034 (8)	−0.0076 (9)	−0.0048 (8)
C20	0.0734 (15)	0.0528 (13)	0.0834 (16)	−0.0018 (11)	−0.0364 (13)	0.0165 (11)
C21	0.0796 (16)	0.0807 (17)	0.0924 (18)	−0.0071 (13)	−0.0594 (15)	0.0143 (14)
C22	0.0561 (12)	0.0641 (13)	0.0659 (13)	−0.0120 (10)	−0.0339 (10)	−0.0019 (10)
C23	0.0822 (16)	0.0438 (11)	0.0838 (16)	−0.0112 (10)	−0.0195 (13)	−0.0091 (11)

### Geometric parameters (Å, º)

**Table:**

S1—O2	1.4161 (16)	C18—C19	1.391 (3)
S1—O1	1.4258 (14)	C19—C20	1.380 (3)
S1—N1	1.6605 (16)	C19—C23	1.500 (3)
S1—C5	1.7571 (19)	C20—C21	1.370 (4)
O3—C8	1.209 (2)	C21—C22	1.377 (4)
N1—C8	1.390 (2)	C1—H1A	0.9600
C1—C2	1.504 (3)	C1—H1B	0.9600
N1—H1	0.8600	C1—H1C	0.9600
C2—C3	1.374 (4)	C3—H3	0.9300
C2—C7	1.374 (3)	C4—H4	0.9300
C3—C4	1.375 (4)	C6—H6	0.9300
C4—C5	1.374 (3)	C7—H7	0.9300
C5—C6	1.374 (3)	C10—H10	0.9300
C6—C7	1.377 (4)	C12—H12	0.9300
C8—C9	1.495 (3)	C13—H13	0.9300
C9—C10	1.341 (2)	C14—H14	0.9300
C9—C17	1.491 (2)	C15—H15	0.9300
C10—C11	1.467 (3)	C16—H16	0.9300
C11—C16	1.389 (3)	C18—H18	0.9300
C11—C12	1.393 (3)	C20—H20	0.9300
C12—C13	1.378 (3)	C21—H21	0.9300
C13—C14	1.364 (4)	C22—H22	0.9300
C14—C15	1.374 (3)	C23—H23A	0.9600
C15—C16	1.384 (3)	C23—H23B	0.9600
C17—C22	1.386 (3)	C23—H23C	0.9600
C17—C18	1.385 (3)		
			
O2—S1—O1	119.71 (8)	C20—C21—C22	120.2 (3)
O2—S1—N1	108.68 (9)	C17—C22—C21	119.9 (2)
O2—S1—C5	109.20 (9)	C2—C1—H1A	110.00
O1—S1—N1	103.40 (8)	C2—C1—H1B	109.00
O1—S1—C5	109.12 (9)	C2—C1—H1C	109.00
N1—S1—C5	105.77 (8)	H1A—C1—H1B	109.00
S1—N1—C8	123.08 (13)	H1A—C1—H1C	109.00
S1—N1—H1	118.00	H1B—C1—H1C	109.00
C8—N1—H1	118.00	C2—C3—H3	119.00
C1—C2—C3	120.9 (2)	C4—C3—H3	119.00
C1—C2—C7	121.6 (2)	C3—C4—H4	120.00
C3—C2—C7	117.5 (2)	C5—C4—H4	120.00
C2—C3—C4	121.8 (2)	C5—C6—H6	120.00
C3—C4—C5	119.5 (2)	C7—C6—H6	120.00
C4—C5—C6	119.8 (2)	C2—C7—H7	119.00
S1—C5—C4	120.43 (17)	C6—C7—H7	119.00
S1—C5—C6	119.78 (15)	C9—C10—H10	115.00
C5—C6—C7	119.5 (2)	C11—C10—H10	115.00
C2—C7—C6	121.9 (2)	C11—C12—H12	119.00
N1—C8—C9	115.33 (15)	C13—C12—H12	119.00
O3—C8—N1	120.79 (17)	C12—C13—H13	120.00
O3—C8—C9	123.88 (17)	C14—C13—H13	120.00
C10—C9—C17	124.16 (16)	C13—C14—H14	120.00
C8—C9—C10	115.24 (16)	C15—C14—H14	120.00
C8—C9—C17	120.42 (15)	C14—C15—H15	120.00
C9—C10—C11	130.45 (17)	C16—C15—H15	120.00
C12—C11—C16	117.83 (18)	C11—C16—H16	120.00
C10—C11—C12	117.32 (17)	C15—C16—H16	120.00
C10—C11—C16	124.79 (17)	C17—C18—H18	119.00
C11—C12—C13	121.2 (2)	C19—C18—H18	119.00
C12—C13—C14	120.0 (2)	C19—C20—H20	119.00
C13—C14—C15	120.0 (2)	C21—C20—H20	119.00
C14—C15—C16	120.4 (2)	C20—C21—H21	120.00
C11—C16—C15	120.46 (19)	C22—C21—H21	120.00
C18—C17—C22	118.86 (17)	C17—C22—H22	120.00
C9—C17—C18	122.21 (16)	C21—C22—H22	120.00
C9—C17—C22	118.87 (17)	C19—C23—H23A	109.00
C17—C18—C19	121.81 (17)	C19—C23—H23B	109.00
C18—C19—C23	121.31 (19)	C19—C23—H23C	109.00
C20—C19—C23	121.30 (19)	H23A—C23—H23B	109.00
C18—C19—C20	117.38 (18)	H23A—C23—H23C	109.00
C19—C20—C21	121.7 (2)	H23B—C23—H23C	110.00
			
O2—S1—N1—C8	51.45 (17)	C17—C9—C10—C11	−4.3 (3)
O1—S1—N1—C8	179.65 (15)	C8—C9—C17—C18	−85.9 (2)
C5—S1—N1—C8	−65.69 (17)	C8—C9—C17—C22	96.9 (2)
O2—S1—C5—C4	−22.48 (19)	C10—C9—C17—C18	99.4 (2)
O1—S1—C5—C4	−155.02 (16)	C10—C9—C17—C22	−77.8 (2)
N1—S1—C5—C4	94.32 (17)	C9—C10—C11—C12	158.7 (2)
O2—S1—C5—C6	156.34 (16)	C9—C10—C11—C16	−24.1 (3)
O1—S1—C5—C6	23.79 (19)	C10—C11—C12—C13	178.6 (2)
N1—S1—C5—C6	−86.87 (18)	C16—C11—C12—C13	1.1 (3)
S1—N1—C8—C9	−175.81 (13)	C10—C11—C16—C15	−177.9 (2)
S1—N1—C8—O3	3.5 (3)	C12—C11—C16—C15	−0.8 (3)
C1—C2—C3—C4	179.8 (2)	C11—C12—C13—C14	−0.4 (4)
C3—C2—C7—C6	1.3 (3)	C12—C13—C14—C15	−0.7 (4)
C1—C2—C7—C6	−179.7 (2)	C13—C14—C15—C16	1.1 (4)
C7—C2—C3—C4	−1.2 (4)	C14—C15—C16—C11	−0.3 (3)
C2—C3—C4—C5	−0.4 (4)	C9—C17—C18—C19	−175.89 (17)
C3—C4—C5—C6	1.8 (3)	C22—C17—C18—C19	1.3 (3)
C3—C4—C5—S1	−179.38 (19)	C9—C17—C22—C21	174.5 (2)
S1—C5—C6—C7	179.47 (17)	C18—C17—C22—C21	−2.8 (3)
C4—C5—C6—C7	−1.7 (3)	C17—C18—C19—C20	1.5 (3)
C5—C6—C7—C2	0.2 (3)	C17—C18—C19—C23	−179.25 (18)
O3—C8—C9—C10	12.7 (3)	C18—C19—C20—C21	−3.0 (3)
O3—C8—C9—C17	−162.47 (18)	C23—C19—C20—C21	177.8 (2)
N1—C8—C9—C10	−168.01 (17)	C19—C20—C21—C22	1.6 (4)
N1—C8—C9—C17	16.8 (2)	C20—C21—C22—C17	1.4 (4)
C8—C9—C10—C11	−179.30 (19)		

### Hydrogen-bond geometry (Å, º)

Cg2 and Cg3 are the centroids of rings C11–C16 and C17–C22, respectively.

**Table:**

*D*—H···*A*	*D*—H	H···*A*	*D*···*A*	*D*—H···*A*
N1—H1···O1^i^	0.86	2.31	3.038 (2)	143
C21—H21···O2^ii^	0.93	2.57	3.468 (4)	163
C16—H16···*Cg*3	0.93	2.88	3.617 (2)	137
C18—H18···*Cg*2^iii^	0.93	2.83	3.646 (2)	168

Symmetry codes: (i) −*x*+2, −*y*+1, −*z*; (ii) −*x*+1, −*y*+1, −*z*; (iii) −*x*+1, −*y*+1, −*z*+1.
